# Chaihu-Shugan-San Decoction Modulates Intestinal Microbe Dysbiosis and Alleviates Chronic Metabolic Inflammation in NAFLD Rats via the NLRP3 Inflammasome Pathway

**DOI:** 10.1155/2018/9390786

**Published:** 2018-07-11

**Authors:** Yinji Liang, Yupei Zhang, Yuanjun Deng, Shu Liang, Yifang He, Yanning Chen, Chan Liu, Chenli Lin, Li Han, Guifang Tu, Qinhe Yang

**Affiliations:** ^1^School of Nursing, Jinan University, No. 601 Huangpu Avenue West, Guangzhou, Guangdong 510632, China; ^2^School of Traditional Chinese Medicine, Jinan University, No. 601 Huangpu Avenue West, Guangzhou, Guangdong 510632, China; ^3^Department of Pathology, School of Basic Medicine, Jinan University, No. 601 Huangpu Avenue West, Guangzhou, Guangdong 510632, China; ^4^Department of Traditional Chinese Medicine, First Affiliated Hospital, Jinan University, No. 613 Huangpu Avenue West, Guangzhou, Guangdong 510632, China

## Abstract

We evaluate the effects of the Chaihu-Shugan-San decoction on intestinal microbe dysbiosis and chronic metabolic inflammation via the NLRP3 pathway in NAFLD rats that were fed a high-fat diet. Twenty-four SD rats (male, six weeks old, 200 ± 20 g) were randomly divided into three groups: normal control group (NC group), high-fat diet-fed group (HFD group), and Chaihu-Shugan-San decoction intervention group (CH group). The NC group rats were given standard feed, the HFD group rats were all fed a high-fat diet (83% standard feed + 10% lard oil + 5% sucrose + 1.5% cholesterol + 0.5% cholate), and the CH group rats were given a HFD plus Chaihu-Shugan-San at 9.6 g•kg^−1^•d^−1^. Body composition, serum and liver lipids, inflammatory markers, intestinal microbial population, and the NLRP3 pathway-associated protein were assessed. The results showed that Chaihu-Shugan-San decoction significantly reduced body weight and total fat mass and the levels of serum LPS, TG, TNF-*α*, IL-1*β*, and IL-18, as well as liver TC, TG, TNF-*α*, IL-1*β*, and IL-18 (*P <* 0.05). The abundance of Enterobacteriaceae (0.375% versus 0.064%,* P* < 0.05), Staphylococcaceae families (0.049% versus 0.016%,* P* < 0.05) and* Veillonella* genus (0.096% versus 0.009%,* P <* 0.01) significantly decreased, whereas the abundance of* Anaeroplasma *genus (0.0005% versus 0.0178%,* P <* 0.01) significantly increased. The expression levels of NLRP3, ASC, and Caspase-1 were changed significantly (*P* < 0.05). In summary, the Chaihu-Shugan-San decoction modulated intestinal microbe dysbiosis, reduced fat accumulation, and alleviated inflammatory factor expression, which are all processes related to the NLRP3 inflammasome pathway in NAFLD rats.

## 1. Introduction

Modern medical studies have suggested that nonalcoholic fatty liver disease (NAFLD) is a manifestation of metabolic syndrome (MS) in the liver that is often associated with obesity, dyslipidemia, and insulin resistance (IR)[1]. In recent years, the incidence and prevalence of NAFLD have increased, and it is one of the most common chronic liver diseases worldwide [2]. In Europe and the United States, the incidence rate of adult NAFLD was 20%-30% [3], whereas the incidence was 15%-30% in China [4]. NAFLD is indicated by more than 5% liver fat accumulation in imaging and/or histological pathology, and 25%-59.10% of NAFLD patients progress to nonalcoholic steatohepatitis (NASH) [5, 6]. NASH is the most serious pathological condition associated with NAFLD, and NASH leads to hepatocyte inflammatory injury, which can develop into cirrhosis or hepatocellular carcinoma (HCC). Despite being viral infection, large amounts of alcohol intake over a long-term period and other causes explain how the majority of cirrhosis cases arise; a small portion of cases still lack a clear cause and are categorized as cryptogenic cirrhosis (CC) [7]. Some studies have found that approximately 50% of CC cases arise from NASH [8], and even a significant proportion of CC patients (15-30%) eventually progress to liver failure and HCC [9, 10]. Furthermore, NAFLD causes or aggravates cardiovascular and cerebrovascular diseases, diabetes, cancer, and other diseases [5].

Inflammation is one of the most important factors in the pathogenesis of NAFLD, and activation of the NLRP3 inflammasome was considered to be the key link to hepatocyte injury, immune cell activation, and expansion of liver inflammation in NAFLD [11]. NLRP3 is currently thought to be activated by various types of pathogen-associated molecular patterns (PAMPs) or damage-associated molecular patterns (DAMPs), especially in hepatocytes and immune cells. Activation of the NLRP3 inflammasome required two links [12]. First, activation of the LPS/TLR4 pathway is the first signal necessary to activate NF-*κ*B, which increases the expression of NLRP3 and IL-1*β* precursors. Second, activation of Caspase-1 and the release of IL-1*β* and IL-18 eventually lead to a “waterfall” inflammatory cascade and cause cell damage. Recent studies have demonstrated that the intestinal microenvironment also plays an important role in NLRP3-mediated liver injury in NAFLD. However, the specific mechanism has not yet been elucidated. Many studies have confirmed that there is a significant difference in the composition and function of the intestinal microbiota between NALFD patients and healthy individuals. Shanab AA et al. [13] found that the prevalence of enteric bacterial overgrowth syndrome (EBOS) in NASH patients was significantly higher than that in healthy controls. It has also been found that the proportion of the* Ruminococcaceae* family of the intestinal microbiota in the fecal samples of NAFLD and NASH patients was lower than that in healthy subjects [14]. In addition, the proportion of* Clostridium coccoides* in adult NASH patients was significantly higher than that in NAFLD patients [15]. It has also been found that the severity of NAFLD was significantly and negatively correlated with the abundance of* γ-Proteus* in the intestine [16]. Although some of the data are controversial, these studies indicated that certain bacteria may be beneficial or harmful to patients with NALFD.

Currently, the prevention and treatment of NAFLD are common in western medicine and traditional Chinese medicine (TCM). New guidelines suggest that health education and lifestyle interventions are still the preferred treatment for NAFLD/NASH. In light of this treatment approach, single herbal medicines, compound TCM and its active ingredients for the prevention, and treatment of NAFLD have become popular topics in this field. It has been found that some TCM compounds, more than 60 single Chinese herbs and 30 active ingredients have good lipid-lowering and liver protection effects [17]. Chaihu-Shugan-San was recorded in the ancient masterpiece “*Jing-Yue book*” as the main treatment for liver depression symptoms. Modern pharmacology has revealed that Chaihu-Shugan-San provides liver protection and has anti-inflammatory properties. Zhang Liangdeng et al. [18] conducted 10 randomized controlled trials (RCTs) with 802 NAFLD subjects, and a meta-analysis showed that the total cholesterol, low-density lipoprotein, alanine aminotransferase (ALT), and aspartate aminotransferase (AST) levels decreased compared with those in a control group following Chaihu-Shugan-San treatment. In addition, a study showed that [19] Chaihu-Shugan-San could improve clinical symptoms and liver function in patients with digestive and metabolic diseases with liver qi stagnation and spleen deficiency syndromes. In an experimental study, preliminary results showed that [20, 21] Chaihu-Shugan-San could repress the inflammatory response and therefore alleviate the progression of NAFLD disease. Therefore, due to the close link between the liver and intestines, our study investigated the effects of Chaihu-Shugan-San on the gut microbiota, SCFA production and its impact on serum and liver lipids, serum LPS, inflammatory markers, and NLRP3 pathways in NAFLD rats.

## 2. Materials and Methods

### 2.1. Animals and Study Design

Twenty-four healthy male SD rats (SPF grade) were purchased from the Experimental Animal Center of Guangzhou University of Traditional Chinese Medicine (license number: SCXK (Guangdong) 2013-0034). After adaptive feeding for one week, all the rats were randomly divided into three groups (n=8): the normal control group (NC group), high-fat diet-fed group (HFD group), and Chaihu-Shugan-San decoction intervention group (CH group). Standard feed and the HFD were derived from the Guangdong Provincial Medical Laboratory Animal Center (license number: SCXK (Guangdong) 2013-0002) using the following HFD processing formula: 83% basal feed + 10% lard oil + 5% sucrose + 1.5% cholesterol + 0.5% cholate. The NC group rats were fed the standard diet, the HFD group rats were fed the HFD, and the CH group rats were fed the HFD + Chaihu-Shugan-San (9.6 g • kg^−1^ • d^−1^) for 16 weeks. Experimental herbal doses were determined using the textbook* Prescriptions of Chinese Materia Medica* (tenth edition) [22] and preliminary research results [23]. All rats from each group had free access to water and were kept in an SPF animal laboratory with a dark and light cycle of 12 hours and temperature range of 18°C-22°C.

### 2.2. Chaihu-Shugan-San Decoction Preparation

All Chaihu-Shugan-San formula granules used in this study were obtained from the same place and the same batch. According to the analysis method described in* China Pharmacopoeia* (2015 edition), the Chaihu-Shugan-San formula granules conformed to the China Pharmacopoeia standards (Committee, 2010). The TCM formula Chaihu-Shugan-San decoction consists of seven herbs, namely, Bupleurum scorzonerifolium Willd., Ligusticum chuanxiong Hort., Citrus aurantium L., Citrus reticulata Blanco, Paeonia lactiflora Pall., Cyperus rotundus L., and Glycyrrhiza uralensis Fisch., which were purchased from Jiangyin Tianjiang Pharmaceutical Co., Ltd. (Jiangsu, China), and the amounts of each herb in one unit of Chaihu-Shugan-San formula in each group are shown in Table 1. According to the amounts of each herb required for one unit of Chaihu-Shugan-San formula, Bupleurum scorzonerifolium Willd. (6 g), Ligusticum chuanxiong Hort. (5 g), Citrus aurantium L. (5 g), Citrus reticulata Blanco (6 g), Paeonia lactiflora Pall. (5 g), Cyperus rotundus L. (5 g), and Glycyrrhiza uralensis Fisch. (3 g) were weighed and then fully dissolved in 2500 mL of ultrapure water heated in a water bath as the stock solution. Then, 14 mL of methanol were added to 10 mL of stock solution, and ultrasonic treatment was conducted for 30 min. Then, the solution was filtered with a 0.25-*μ*m microporous membrane at room temperature.

### 2.3. HPLC Analysis of the Major Components in the Chaihu-Shugan-San Decoction

To confirm the major components of the Chaihu-Shugan-San decoction used in this study, high-performance liquid chromatography (HPLC) analysis was performed. Briefly, chromatographic separation was performed on a COSMOSIL Packed Column 5C18-MS-II (4.6 mm × 250 mm) using acetonitrile as the mobile phase A, trifluoroacetic acid (TFA) as the mobile phase B, and detection wavelengths of 210 and 235 nm, respectively. Saikosaponin A was detected at a wavelength of 210 nm, and ferulic acid, neohesperidin, naringin, hesperidin, paeoniflorin, liquiritin, and glycyrrhizic acid were detected at 235 nm.

### 2.4. EchoMRI™ Analysis of Whole-Body Composition

Total body fat, lean mass, free water, and total body water content in vivo were evaluated using an EchoMRI 2012 (EchoMRI, Houston, TX, USA) and quantitative magnetic resonance body composition analyzers. These tests were conducted at week 16 prior to sacrifice as previously described [24].

### 2.5. Biochemical Measurements of Lipids and Inflammatory Markers in Serum and Liver Tissue

Blood plasma (3-5 mL) was centrifuged at 3000 rpm and 4°C for 10 min, and the upper layer of the serum was collected in a 1.5-mL EP tube. Hepatic tissue (0.1 g) was placed in isopropanol (0.9 mL) and homogenized with a tissuelyser-II homogenizer, followed by centrifugation at 3500 rpm and 4°C for 10 min. The clear supernatants were collected, and the levels of TC and TG in serum and liver tissue were determined with an automatic biochemical analyzer. Serum and liver TNF-*α*, IL-1*β*, and IL-18 levels were quantitatively detected using an enzyme-linked immunosorbent assay. Serum LPS in the portal vein was determined with an endpoint chromogenic assay (No.160525, Xiamen Bioendo Technology, Co., Ltd., Fujian, China).

### 2.6. Western Blot Analyses

Approximately 200 mg of liver tissue was added to 2 mL of RIPA lysate and 20 *μ*L of protease inhibitor. The ultrasonic homogenizer was used to homogenize the sample in an ice bath until no granules were detected. After centrifugation, the middle layer of the liquid was collected (the upper layer was oil, and the lower layer had a little precipitation), and centrifugation was repeated one time. For the BCA method and protein hyperthermia degeneration, the cells were transferred to a PVDF membrane and 5% skim milk powder at room temperature for 1.5 h. The membrane was immersed in the TLR4 antibody 1:500, NLRP3 antibody 1:500, ASC antibody 1:300, Caspase-1 antibody 1:500, or NF-*κ*B p65 antibody 1:1000 at 4°C overnight. After washing the membrane, goat anti-rabbit IgG (H+L), mouse/human ads-HRP (1:20,000; Southern Biotech, 4050-05), or rabbit anti-mouse IgG (H+L)-HRP (1:10000; Southern Biotech, 6170-05) was incubated with the membrane for 1.5 h at room temperature. After washing the film, ECL luminescence solution was added, and the film was developed. The semiquantitative analysis of the bands was performed using software. The OD value of the target protein was divided by the OD value of the *β*-actin band as the final result.

### 2.7. Sequencing Analyses of Intestinal Microbiota

At 1-2 d before euthanasia, fresh fecal samples were collected under anaerobic conditions and immediately frozen in liquid nitrogen at the end of the 16-week intervention period. The genomic DNA of each fecal sample was extracted with QIAamp DNA mini kits (Qiagen, Valencia, CA, USA), and bacterial genome DNA was detected by gel electrophoresis. The sample DNA was amplified to enrich the V3-V4 region of 16S rDNA genes in bacteria with a specific primer containing a barcode sequence [25]. Then, the PCR amplification product was recovered and quantified using a QuantiFluor™ fluorometer. The purified amplification products were mixed in equal amounts and connected through sequencing joints. Then, a sequencing library was built, and sequencing with Hiseq2500 PE250 was conducted.

### 2.8. Gas Chromatography Analysis of Short-Chain Fatty Acids

At the end of the intervention period for 16 weeks, the rats were deprived of food for 12 h, weighed, and injected with 2% sodium pentobarbital solution (8 mg/kg). The contents in the large intestine were frozen immediately after collection at the end of treatment and stored in liquid nitrogen until used. Standard reference materials for acetic acid, propionic acid, isobutyric acid, butyric acid, isovaleric acid, valeric acid, and 2-ethylbutyric acid (chromatographic grade) were obtained from the Aladdin Company in China. The 5 M HCL, diethyl ether, and methanol (analytical grade) were purchased from Guangzhou Biological Technology Co., Ltd. (Guangdong, China). Gas chromatography (GC) was carried out for analysis of stool extracts as previously described [20]. Briefly, one gram of flesh-frozen stool samples was extracted with at least 5 mL of deionized water, mixed for approximately 5 min, and brought up to a final suspension concentration of 17% (w/w). The 2-methylbutanoic acid stock solution (200 mM, 25 *μ*l) was added to the suspension at a final concentration of 1 mM. The tube was centrifuged for 5 min in a tabletop centrifuge, and the supernatant was collected and mixed with 5 M HCL to adjust the pH value to 2-3. The hexane layer was placed in a GC vial, and the vial was then capped and stored at −20°C until GC analysis.

### 2.9. Statistical Analyses

Statistical analyses were performed using SPSS 19.0. Data are expressed as the mean ± SEM. Differences were analyzed by one-way ANOVA and adjusted by Bonferroni correction to counteract the problem of multiple comparisons.* P* values < 0.05 were considered significant.

## 3. Results

### 3.1. The Major Components of the Chaihu-Shugan-San Decoction

**Figure 1:**
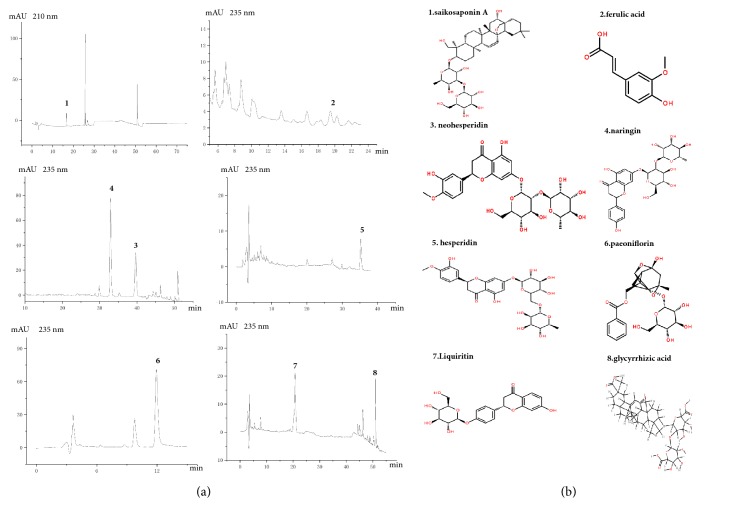
The herbal formula Chaihu-Shugan-San. (a) HPLC graphs of the Chaihu-Shugan-San decoction. High-performance liquid chromatography (HPLC) was performed to identify the phytochemical profiles of the Chaihu-Shugan-San decoction. (b) The main chemicals in the Chaihu-Shugan-San decoction were the following: 1, saikosaponin A; 2, ferulic acid; 3, neohesperidin; 4, naringin; 5, hesperidin; 6, paeoniflorin; 7, liquiritin; and 8, glycyrrhizic acid.

The Chaihu-Shugan-San decoction containing Bupleurum scorzonerifolium Willd., Ligusticum chuanxiong Hort., Citrus aurantium L., Citrus reticulata Blanco, Paeonia lactiflora Pall., Cyperus rotundus L., and Glycyrrhiza uralensis Fisch. were prepared using the procedure in Section 2.2. The sample solution was prepared separately, and HPLC conditions were measured for eight components using the procedure in Section 2.3. The results are shown in Figure 1.

### 3.2. The Chaihu-Shugan-San Decoction Modulates the Intestinal Microbiota in NAFLD Rats

**Figure 2:**
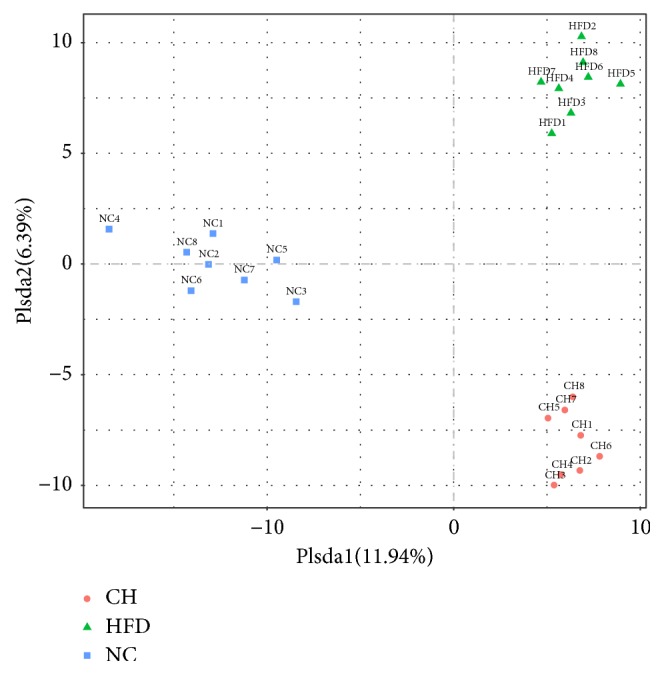
OTU principal component analysis (PCA). The vertical and ordinate are the first and second principal components, and the percentage in parentheses indicates the contribution of the principal component to the sample difference. The points on the graph indicate the individual samples. Different colors represent samples belonging to different groups.

**Figure 3:**
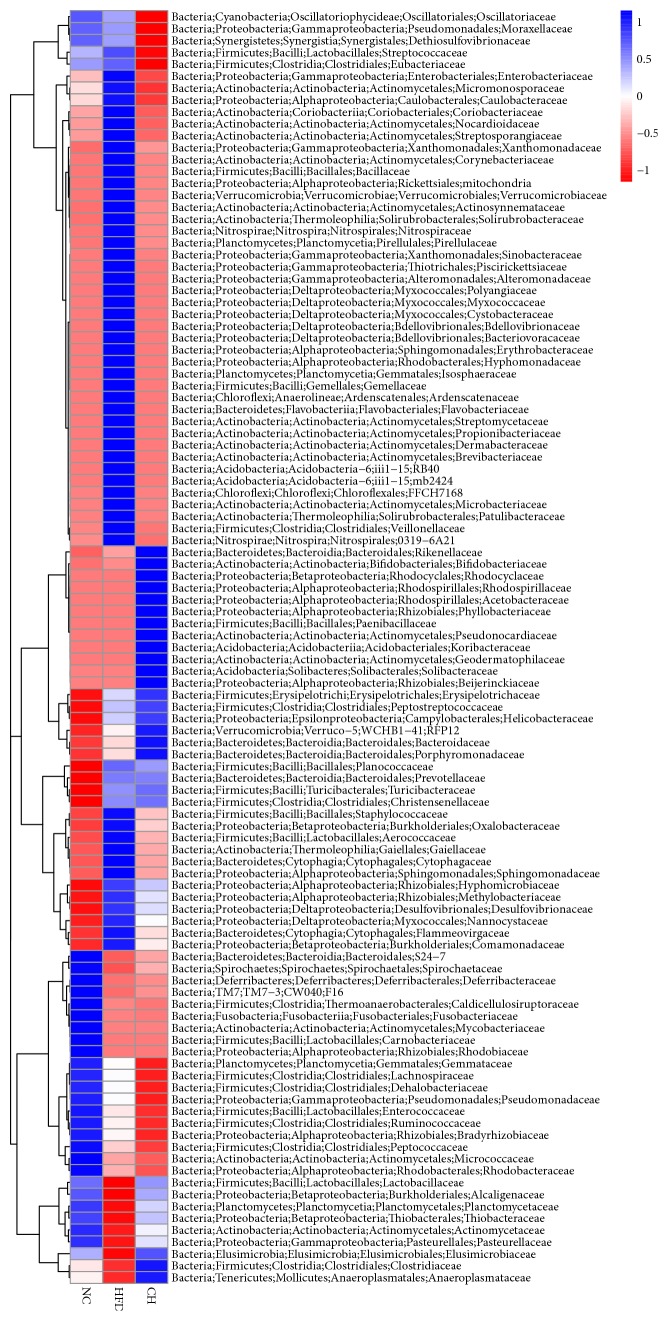
Heat map of bacteria with changes between each two groups at the family level. Hierarchical clustering with a heat map shows the relative abundance of representative OTUs (i.e., samples with the greatest difference among the three group means from each family) selected due to differences corresponding to* P <* 0.05, obtained with differential expression analysis of the three groups. The OTUs are shown as phylum, class, order, and family.

**Figure 4:**
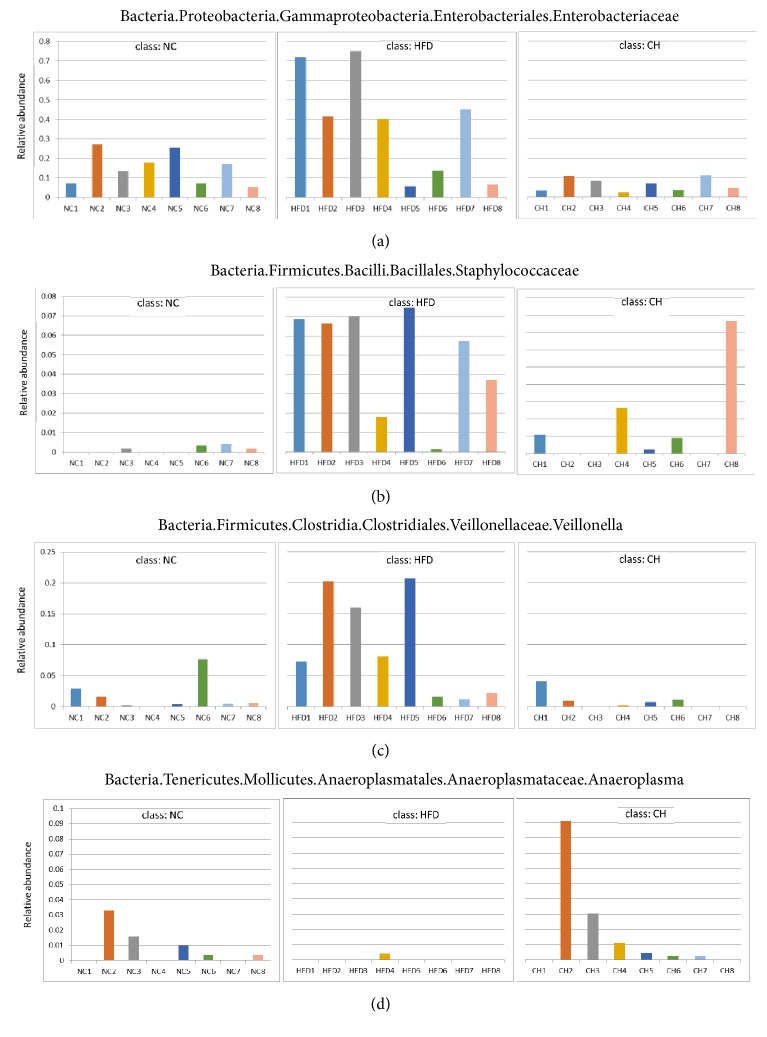
The actual representation of individual species in the comparison group NC, HFD, and CH. (a) Enterobacteriaceae, (b) Staphylococcaceae, (c) Veillonella, and (d) Anaeroplasma, n=8.

Based on the relative abundance of operational taxonomic units (OTUs), principal component analysis (PCA) showed that the sets of fecal samples in the NC, HFD, and CH groups are clearly separated, which indicates that the structures of the intestinal microbiota in these three groups were partially different (Figure 2). Furthermore, the relative abundance of family taxa had a* P* value of < 0.05 according to the differential expression analysis (nonparametric ANOVA with false discovery rate [FDR] correction), which was expressed as a heat map (Figure 3), including hierarchical clustering (HCN). At the family level, the abundances of 44 strains in the HFD group were different compared with those in the NC group, especially for F16, Lactobacillaceae, Clostridiaceae, and Enterobacteriaceae (*P* < 0.05). Compared with the HFD group, 18 Bacteriaceae in the CH group were different, in particular the abundances of Enterobacteriaceae (0.375% versus 0.064%,* P <* 0.05) and Staphylococcaceae (0.049% versus 0.016%,* P <* 0.05) were significantly different (Figures 4(a) and 4(b)). At the genus level, compared with the NC group, the abundances of 55 strains in the HFD group were different, especially* Veillonella, Anaeroplasma *and* Cupriavidus* (*P* < 0.05). Compared with the HFD group, the abundance of 27 strains, such as* Veillonella* (0.096% versus 0.009%,* P <* 0.01) and* Anaeroplasma* (0.0005% versus 0.0178%,* P <* 0.01), significantly changed (*P* < 0.05) (Figures 4(c) and 4(d)). Furthermore, compared with the NC group, the isobutyric acid and isovaleric acid levels in feces of the HFD group rats reduced by 35.6% and 34.5%, respectively (*P* < 0.01). While compared with the HFD group, the butyric acid showed a tendency towards increase (*P *= 0.074). But the differences of other SCFA in dry feces were not significant between the three groups (Table 2).

### 3.3. The Chaihu-Shugan-San Decoction Reduced Bodyweight and Total Body Fat Content in NAFLD Rats

**Figure 5:**
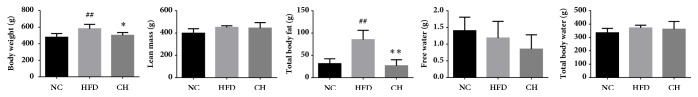
Body weight, total body fat, lean mass, free water, and total body water in three SD rat groups fed a control diet or a HFD supplemented with Chaihu-Shugan-San for 16 weeks. ^#^*P <* 0.05 NC versus HFD, ^##^*P <* 0.01 NC versus HFD, ^*∗*^*P <* 0.05 HFD versus CH, ^*∗∗*^*P <* 0.01 HFD versus CH, n=6-8.

Furthermore, body composition was assessed by EchoMRI analysis. Compared with the NC group, the body weight (g) and total body fat mass (g) of the HFD group significantly increased (*P <* 0.01). These indices of the CP group were all significantly lower than those of the HFD group (*P* < 0.05 or* P* < 0.01). The results showed that Chaihu-Shugan-San significantly reduced HFD-induced weight gain and body fat accumulation (Figure 5, Supplemental Table 1).

### 3.4. The Chaihu-Shugan-San Decoction Reduced Lipid Accumulation and Chronic Low-Grade Inflammation in NAFLD Rats

**Figure 6:**
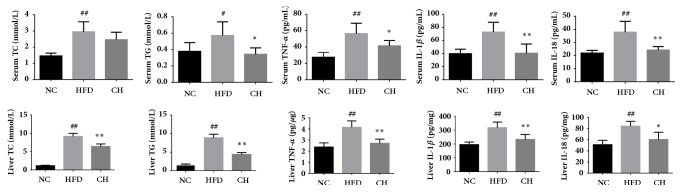
Serum and liver TC, TG, TNF-*α*, IL-1*β*, and IL-18 in three groups: SD rats fed a control diet or a HFD supplemented with Chaihu-Shugan-San for 16 weeks. ^#^*P <* 0.05 NC versus HFD, ^##^*P <* 0.01 NC versus HFD, ^*∗*^*P <* 0.05 HFD versus CH, and ^*∗∗*^*P <* 0.01 HFD versus CH, n=6.

Compared with the NC group, concentrations of TC, TG, TNF-*α*, IL-1*β*, and IL-18 in the serum and liver tissue homogenates of the HFD group significantly increased (*P* < 0.05 or* P* < 0.01). In addition to serum TC, all of the above indicators significantly decreased (*P* < 0.05 or* P* < 0.01). The results indicated the presence of severe lipid deposition and metabolic inflammation and that Chaihu-Shugan-San reduced the levels of liver fat and inflammatory cytokines in NAFLD rats (Figure 6, Supplemental Table 2).

### 3.5. Chaihu-Shugan-San Decoction Influenced the Protein Expression of the NLRP3 Pathway in NAFLD Rats

**Figure 7:**
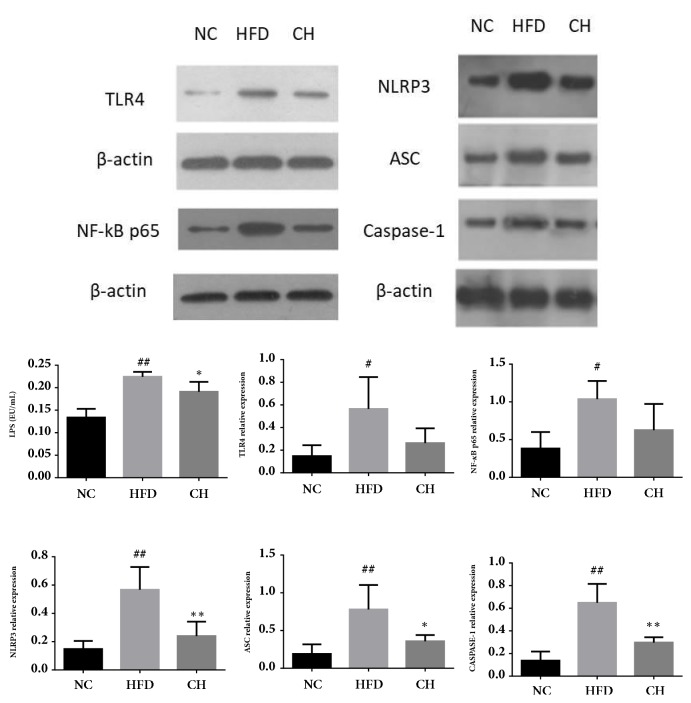
Serum LPS and protein expression of TLR4, NLRP3, ASC, Caspase-1, and NF-kB p65 in SD rats fed a control diet or a HFD supplemented with Chaihu-Shugan-San for 16 weeks. These proteins were quantified via western blotting in the hepatic tissue. ^#^*P <* 0.05 NC versus HFD, ^##^*P <* 0.01 NC versus HFD, ^*∗*^*P <* 0.05 HFD versus CH, and ^*∗∗*^*P <* 0.01 HFD versus CH, n=4.

Compared with the NC group, the serum LPS levels and the expression of TLR4, NLRP3, ASC, Caspase-1, and NF-kB p65 protein in the HFD group significantly increased (*P* < 0.05 or* P* < 0.01). Compared with the HFD group, serum LPS and hepatic NLRP3, ASC, and Caspase-1 were lowered by Chaihu-Shugan-San (*P* < 0.05 or* P* < 0.01) (Figure 7, Supplemental Table 3).

## 4. Discussion

The Chinese herbal compound Chaihu-Shugan-San is composed of Bupleurum scorzonerifolium Willd., Ligusticum chuanxiong Hort., Citrus aurantium L., Citrus reticulata Blanco, Paeonia lactiflora Pall., Cyperus rotundus L., and Glycyrrhiza uralensis Fisch.. Chaihu-Shugan-San has a wide range of clinical applications, especially for the treatment of depression [26], hyperlipidemia, NAFLD [27], and some digestive diseases [28]. The efficacy of the Chinese herbal compound was determined according to the composition and content of the compound. In our experiments, the chemical compositions of Bupleurum scorzonerifolium Willd., Ligusticum chuanxiong Hort., Citrus aurantium L., Citrus reticulata Blanco, Paeonia lactiflora Pall., Cyperus rotundus L., and Glycyrrhiza uralensis Fisch. extracts were analyzed by HPLC. The results showed that the fingerprints, including saikosaponin A, ferulic acid, neohesperidin, naringin, hesperidin, paeoniflorin, liquiritin, and glycyrrhizic acid, were consistent with the reference substance. Modern pharmacodynamics also revealed that Chaihu-Shugan-San contains a variety of chemical components, which mainly include saponins, flavonoids, terpenoids, and phenolic acids. These ingredients were directly related to the many pharmacological effects of the compound. It was found that saikosaponin A and saikosaponin D have anti-inflammatory effects [29–31] and regulate lipid metabolism [32]. Li R. believed that saikosaponin could inhibit the release of inflammatory mediators and granuloma growth and inhibit a 5-HT-induced capillary permeability increase, as well as significantly inhibiting leukocyte migration and connective tissue hyperplasia [33]. Tetramethylpyrazine, which is the active component of Ligusticum chuanxiong Hort., has anti-inflammatory effects [34], whereas ferulic acid regulates lipid metabolism by inhibiting lipid oxidation and lowering cholesterol levels in blood lipids [35]. Hesperidin, naringin, and paeoniflorin, which are the active ingredients of Citrus aurantium L. and Paeonia lactiflora Pall., also have anti-inflammatory effects [36–38] and regulate lipid metabolism [39, 40]. The study by Wang W. et al. [41] showed that hesperidin may inhibit the expression and activity of COX-2 in liver tissue, and, therefore, it had a therapeutic effect on NAFLD rats. It could be seen that the active ingredients of Chaihu-Shugan-San had obvious anti-inflammatory, antioxidative, and lipid metabolism regulatory effects, but the exact target of this compound needs to be further studied.

It has been considered that the intestinal microbiota produces some toxic components that may potentially harm the liver. These bacterial toxic components reach the liver through the portal vein, activate liver Kupffer cells, and then stimulate the production of NO and cytokines, which is a process known as intestinal endotoxemia. Production of endogenous ethanol and LPS could induce intestinal macrophage activation, which stimulates the secretion of proinflammatory cytokines and may be one of the possible intestinal microbiota mechanisms affecting intestinal barrier function [42]. In all bacterial products, LPS appeared to be the most important in the pathogenesis of NAFLD. LPS is the active ingredient of endotoxins, especially when it is combined with lipopolysaccharide-binding protein (LBP), CD14, TLR4, and lymphocyte antigen 96. Endogenous LPS is continuously produced by the death of intestinal gram-negative bacteria and then migrates into the liver through TLR4-mediated signaling pathways. Therefore, changes in intestinal permeability could promote endotoxins in the liver. It has been found that TLR4 works primarily in a MyD88-dependent pathway. TLR4 binds to CD14 on the surface of cell membranes, initiates LPS-induced signal transduction, and activates NF-*κ*B and the downstream proinflammatory cytokines TNF and cyclooxygenase 2[43]. A study was conducted to establish a model of fatty liver in rats using intravenous nutrition, and polymyxin was administered to inhibit the activity of endogenous LPS. The results showed that TNF production, liver fat, and TG deposition were remarkably reduced by polymyxin [44]. Our study revealed that, compared with the NC group, the expression of TLR4 and NF-*κ*B p65 in the liver tissue of the HFD group significantly increased (*P* < 0.05), whereas the Chaihu-Shugan-San mildly counteracts these effects. Accompanied by an increase in serum LPS, this increase showed that HFD promotes intestinal microbiota imbalance and increasingly severe intestinal endotoxemia. Furthermore, LPS may induce TLR4 pathway-related protein activation and the onset of NAFLD.

As previously mentioned, activation of the NLRP3 inflammasome is considered to be a key link in TLR4-mediated hepatocyte injury, immune cell activation, and expansion of liver inflammation in NAFLD [11]. The activation of the NLRP3 inflammasome and the release of downstream IL-1*β* and IL-18 have been shown to be associated with the pathogenesis of various liver diseases [45]. Currently, the focus of this field is on the effects of dietary and intestinal microflora interactions on the activation of inflammatory mediators in the liver [46]. Another study found that mature IL-1*β* could enhance the immune signals of liver immune cells, which leads to upregulation of monocyte chemoattractant protein-1 (MCP-1) and TNF-*α* expression. MCP-1 aggravated hepatocellular steatosis, while IL-1*β* was more sensitive to TNF-induced hepatocellular toxicity. In addition, the proinflammatory effects of IL-1*β* may be synergistic with TLR4 signaling and significantly enhance LPS-induced release of inflammatory cytokines [47]. In our study, we found increased expression of the NLRP3 inflammasome in NAFLD rats, and Chaihu-Shugan-San significantly inhibited overexpression of the NLRP3 inflammasome. This result suggested that NLRP3 may be a therapeutic target of Chaihu-Shugan-San.

Additionally, we found that the structure of the intestinal microflora in NAFLD rats was partially reconstructed in HFD-fed NAFLD rats, and the enrichment and diversity of intestinal microflora changed after Chaihu-Shugan-San treatment for 16 weeks. At the family level, we found that the abundance of Enterobacteriaceae decreased by nearly 90% in NAFLD rats (*P* < 0.05) compared with that in the NC group rats. Enterobacteriaceae is a major bacterium among the gram-negative bacilli, which include some conditional pathogens, such as* Escherichia coli, Salmonella, Shigella, *and* Klebsiella*, which are an important source of LPS. A recent RCT showed that aerobic bacteria, such as* Enterococcus faecalis *and Enterobacteriaceae, were significantly increased in the NASH group and that anaerobes, such as* Bacillus bifidus* and* Lactobacillus*, were reduced compared with the amount in the healthy group [48]. In this study, Chaihu-Shugan-San significantly reduced the abundance of Enterobacteriaceae and the concentration of serum LPS. It is likely that one of the intestinal mechanisms of Chaihu-Shugan-San alleviated intestinal endotoxemia in NAFLD rats. In addition, at the genus level, we were surprised to find that the abundance of* Veillonella* increased significantly in the HFD group rats, and* Anaeroplasma* decreased significantly. Currently, the* Veillonella *genus is believed to produce SCFAs through the succinic acid metabolic pathway, and the increase in the abundance of the* Veillonella* genus often leads to an increase in intestinal SCFA concentrations [49]. Compared with those in the NC group, this study found that isobutyric acid and isovaleric acid levels of dry feces in the HFD group were significantly reduced. But the differences of other SCFA in dry feces were not significant among the three groups. Therefore, the relationship between SCFAs and NAFLD needs further investigation.

## 5. Conclusions

In summary, the Chaihu-Shugan-San decoction modulated intestinal microbe dysbiosis, reduced body fat and intrahepatic fat accumulation and alleviated LPS-induced endotoxemia and inflammatory factor expression, which are all processes related to the NLRP3 inflammasome in NAFLD rats.

## Figures and Tables

**Table 1:** The Chaihu-Shugan-San decoction of each dose.

Chinese name	Common name	Botanical name	Part used	Weight (g)
Chaihu	Bupleuri Radix	Bupleurum scorzonerifolium Willd.	Root	6
Chuanxiong	Chuanxiong Rhizoma	Ligusticum chuanxiong Hort.	Rhizome	5
Zhiqiao	Aurantii Fructus	Citrus aurantium L.	Fruit	5
Chenpi	Citri Reticulatae pericarpium	Citrus reticulata Blanco	Pericarp	6
Baishao	Paeoniae Radix Alba	Paeonia lactiflora Pall.	Root	5
Xiangfu	Cyperi Rhizoma	Cyperus rotundus L.	Rhizome	5
Zhigancao	Glycyrrhizae Radix et Rhizoma	Glycyrrhiza uralensis Fisch., Glycyrrhiza inflata Bat. or Glycyrrhiza glabra L.	Root and rhizome	3

**Table 2:** Mean SCFA (mmol/L) concentrations in dry feces of experimented rats.

Category	acetic acid	propionic acid	isobutyric acid	butyric acid	isovaleric acid	valeric acid
NC group	0.269±0.116	0.241±0.052	0.045±0.011	0.438±0.106	0.087±0.014	0.064±0.009
HFD group	0.294±0.105	0.336±0.112	0.029±0.009 ^**##**^	0.373±0.151	0.057±0.009 ^**##**^	0.048±0.025
CH group	0.389±0.183	0.352±0.119	0.032±0.008	0.393±0.114	0.044±0.016	0.047±0.008

^##^ Significant difference compared with NC group rats in fecal SCFAs based on ANOVA as described in the Methods. *P <* 0.01 NC versus HFD, n=8.

## Supplementary Materials

Supplementary Materials Supplemental Table 1: Body weight and body composition. (DOCX) Supplemental Table 2: Lipids and inflammatory cytokines in serum and liver. (DOCX) Supplemental Table 3: The relative expression of TLR4, NLRP3, ASC, Caspase-1, NF-kB p65. (DOCX)
