# Supplementary material for: Chaihu-Shugan-San Decoction Modulates Intestinal Microbe Dysbiosis and Alleviates Chronic Metabolic Inflammation in NAFLD Rats via the NLRP3 Inflammasome Pathway
Source: Evid Based Complement Alternat Med. 2018 Jul 11;2018:9390786. doi: 10.1155/2018/9390786 (PMC6076928; doi:10.1155/2018/9390786)
Supplement: Supplementary Materials — Supplemental Table 1: Body weight and body composition. (DOCX) Supplemental Table 2: Lipids and inflammatory cytokines in serum and liver. (DOCX) Supplemental Table 3: The relative expression of TLR4, NLRP3, ASC, Caspase-1, NF-kB p65. (DOCX) [file 9390786.f1.docx]

**Supplementary Materials**

**Supplemental Table 1:** Body weight and body composition

|  | NC | HFD | CH |
| --- | --- | --- | --- |
| Body weight (g) | 478.2±44.9 | 581.3±53.2^##^ | 501.5±34.0^**^ |
| Total body fat (g) | 31.34±11.0 | 85.23±20.97^##^ | 26.70±13.52^*^ |
| Lean mass (g) | 398.1±41.04 | 451.3±13.88 | 444.2±49.52 |
| Free water (g) | 1.403±0.403 | 1.183±0.499 | 0.850±0.430 |
| Total body water (g) | 334.9±33.06 | 371.2±20.54 | 361.8±58.05 |

^#^*P <* 0.05 NC vs HFD, ^##^*P <* 0.01 NC vs HFD, ^*^ *P <* 0.05 HFD vs CH, ^**^ *P <* 0.01 HFD vs CH, n=6-8.

**Supplemental Table 2:** Lipids and inflammatory cytokines in serum and liver

|  | NC | HFD | CH |
| --- | --- | --- | --- |
| Serum TC (mmol/L) | 1.46±0.18 | 2.95±0.62^##^ | 2.46±0.47 |
| Serum TG (mmol/L) | 0.38±0.11 | 0.57±0.17^#^ | 0.34±0.08^*^ |
| Serum TNF-α(pg/mL) | 27.50±5.93 | 56.38±12.63^##^ | 41.39±6.65^*^ |
| Serum IL-1β (pg/mL) | 39.81±6.81 | 72.85±14.94^##^ | 40.49±13.97^**^ |
| Serum IL-18 (pg/mL) | 21.84±2.09 | 37.86±8.25^##^ | 24.19±2.66^**^ |
| Portal vein serum LPS (EU/mL) | 0.13±0.02 | 0.22±0.01^##^ | 0.19±0.02^*^ |
| Liver TC (mmol/L) | 1.14±0.14 | 9.16±0.81^##^ | 6.42±0.67^**^ |
| Liver TG (mmol/L) | 1.52±0.28 | 8.89±0.94^##^ | 4.38±0.52^**^ |
| Liver TNF-α(pg/ug) | 2.37±0.37 | 4.15±0.58^##^ | 2.71±0.38^**^ |
| Liver IL-1β (pg/mg) | 195.64±18.15 | 317.39±40.30^##^ | 233.29±35.09^**^ |
| Liver IL-18 (pg/mg) | 51.11±8.02 | 84.62±8.66^##^ | 60.45±13.23^*^ |

^#^*P <* 0.05 NC vs HFD, ^##^*P <* 0.01 NC vs HFD, ^*^ *P <* 0.05 HFD vs CH, ^**^ *P <* 0.01 HFD vs CH, n=6.

**Supplemental Table 3:** The relative expression of TLR4, NLRP3, ASC, Caspase-1, NF-kB p65

|  | NC | HFD | CH |
| --- | --- | --- | --- |
| TLR4 | 0.148±0.096 | 0.565±0.282^#^ | 0.263±0.130 |
| NLRP3 | 0.148±0.057 | 0.567±0.161^##^ | 0.239±0.102^**^ |
| ASC | 0.190±0.126 | 0.780±0.325^##^ | 0.358±0.083^*^ |
| CASPASE-1 | 0.138±0.081 | 0.649±0.167^##^ | 0.297±0.046^**^ |
| NF-kB p65 | 0.382±0.218 | 1.037±0.242^#^ | 0.625±0.376 |

^#^*P <* 0.05 NC vs HFD, ^##^*P <* 0.01 NC vs HFD, ^*^ *P <* 0.05 HFD vs CH, ^**^ *P <* 0.01 HFD vs CH, n=4.
